# Explainable fuzzy clustering framework reveals divergent default mode network connectivity dynamics in schizophrenia

**DOI:** 10.3389/fpsyt.2024.1165424

**Published:** 2024-02-15

**Authors:** Charles A. Ellis, Robyn L. Miller, Vince D. Calhoun

**Affiliations:** ^1^ Wallace H. Coulter Department of Biomedical Engineering, Georgia Institute of Technology and Emory University, Atlanta, GA, United States; ^2^ Tri-institutional Center for Translational Research in Neuroimaging and Data Science, Georgia State University, Atlanta, GA, United States; ^3^ Georgia Institute of Technology, Emory University, Atlanta, GA, United States; ^4^ Department of Computer Science, Georgia State University, Atlanta, GA, United States

**Keywords:** explainable AI, fuzzy clustering, dynamical functional network connectivity, resting state functional magnetic resonance imaging, schizophrenia, default mode network

## Abstract

**Introduction:**

Dynamic functional network connectivity (dFNC) analysis of resting state functional magnetic resonance imaging data has yielded insights into many neurological and neuropsychiatric disorders. A common dFNC analysis approach uses hard clustering methods like k-means clustering to assign samples to states that summarize network dynamics. However, hard clustering methods obscure network dynamics by assuming (1) that all samples within a cluster are equally like their assigned centroids and (2) that samples closer to one another in the data space than to their centroids are well-represented by their centroids. In addition, it can be hard to compare subjects, as in some cases an individual may not manifest a state strongly enough to enter a hard cluster. Approaches that allow a dimensional approach to connectivity patterns (e.g., fuzzy clustering) can mitigate these issues. In this study, we present an explainable fuzzy clustering framework by combining fuzzy c-means clustering with several explainability metrics and novel summary features.

**Methods:**

We apply our framework for schizophrenia (SZ) default mode network analysis. Namely, we extract dFNC from individuals with SZ and controls, identify 5 dFNC states, and characterize the dFNC features most crucial to those states with a new perturbation-based clustering explainability approach. We then extract several features typically used in hard clustering and further present a variety of unique features specially designed for use with fuzzy clustering to quantify state dynamics. We examine differences in those features between individuals with SZ and controls and further search for relationships between those features and SZ symptom severity.

**Results:**

Importantly, we find that individuals with SZ spend more time in states of moderate anticorrelation between the anterior and posterior cingulate cortices and strong anticorrelation between the precuneus and anterior cingulate cortex. We further find that individuals with SZ tend to transition more rapidly than controls between low-magnitude and high-magnitude dFNC states.

**Conclusion:**

We present a novel dFNC analysis framework and use it to identify effects of SZ upon network dynamics. Given the ease of implementing our framework and its enhanced insight into network dynamics, it has great potential for use in future dFNC studies.

## 1 Introduction

Resting state functional magnetic resonance imaging (rs-fMRI) dynamic functional network connectivity (dFNC) data has historically been used to give insight into a variety of neurological (1) and neuropsychiatric disorders (2–6) and cognitive functions (7, 8). A common dFNC analysis approach involves applying a hard clustering approach (e.g., k-means clustering) to assign dFNC samples to a set of dynamical states that are supposedly representative of the overall time series (2, 9–15). Features can then be extracted based on the identified time-resolved states that can give insight into various aspects of the state dynamics. This method has been widely applied but makes a critical assumption that could obscure useful disease-related dynamics. Namely, it assumes that all samples assigned to a state equally belong to a state. This is problematic given that samples very near to one another in the data space may be assigned to two distant cluster centroids. A few studies have presented fuzzy clustering approaches that indicate the degree to which samples belong to different states, but this area is still highly understudied (11, 15). Although explainability methods have been developed specifically to help characterize identified states, the use of hard clustering methods can make explainability difficult. In this study, we present a novel explainable fuzzy clustering framework for fMRI dFNC that identifies fuzzy states and assigns samples a probability of belonging to each state. We further present novel dynamical features that use the output probabilities and demonstrate their utility by applying them to identify differences in the dynamics of default mode network (DMN) activity between individuals with schizophrenia (SZs) and healthy controls (HCs).

Several modalities have been used for insight into the effects of neurological and neuropsychiatric disorders upon brain dynamics. These include electroencephalography (EEG), magnetoencephalography (MEG), and fMRI. All three modalities – EEG (16–18), MEG (19), and fMRI (13, 20–24) - have been used extensively for SZ analysis. EEG and MEG capture much higher resolution temporal information. However, localizing the region of the brain associated with MEG and EEG signals can be challenging. In contrast, fMRI has much higher spatial resolution at a lower temporal resolution relative to EEG and fMRI. A select group of studies have also combined multimodal EEG, MEG, or fMRI data for insight into disorders (9). However, in general, fMRI is more often used in SZ analysis than the other modalities. Within fMRI analysis, both task (25, 26) and resting state (10, 12, 13, 27–32) data are frequently used. However, resting state data offers several advantages. Specifically, the majority of brain activity is spontaneous (i.e., better captured by resting state), so resting state analysis provides an avenue to understand how the brain operates under most circumstances (32). Additionally, task performance in healthy controls relative to individuals with schizophrenia often varies greatly, introducing a potential confounder into any neuroimaging analyses (32).

Many studies have analyzed resting state fMRI data within the context of SZ and other neuropsychiatric disorders. For example, studies have used independent components (ICs) (24), spectral features (9, 33), and functional network connectivity. Functional network connectivity offers a unique benefit in that it provides insights into the interaction of different brain regions and networks. Early in the use of rs-fMRI functional network connectivity, it was more common to analyze the correlation between brain regions across a whole recording. This approach is referred to as static functional network connectivity (sFNC) (34). Although sFNC had widespread use, a number of studies found that dFNC (i.e., functional connectivity captured in windows over time) offered insights into brain interactions that would otherwise be obscured by sFNC analysis (32, 34). Both sFNC and dFNC have been used to gain insight into a variety of neurological and neuropsychiatric disorders and cognitive functions, including Alzheimer’s disease (1, 35, 36), major depressive disorder (3–6), schizophrenia (2, 37), cognition (7), and spatial orientation (8). However, dFNC offers greater opportunities to learn about the brain than sFNC. As discussed in (32, 34), early studies extracted functional network connectivity from time-series extracted using one seed per brain region, multiple seeds from a given region of interest (ROI), multiple seeds from subregions of multiple ROIs, and seeds from whole-brain ROIs. However, in recent years, the fully automated, group independent component analysis (ICA)-based Neuromark pipeline has been developed as a approach for extracting time-series used to calculate functional network connectivity (38). It yields components that are reproducible across datasets and studies and contains components from a variety of brain networks and subregions. Furthermore, it has been used in many studies (1, 3, 24, 35–42).

Multiple approaches have been used to analyze dFNC extracted using the NeuroMark pipeline or other approaches. Many studies have used classification approaches (21, 22, 43) or a combination of clustering and classification (39). However, a described in (32), a many studies have used clustering approaches (9–15). These clustering approaches involve assigning dFNC samples to states that summarize the dFNC time-series. Features can then be extracted to quantify various aspects of the state time-series. By far the most common clustering approach used for identifying states of dFNC activity is k-means clustering (44). K-means clustering involves randomly initializing cluster centroids, calculating the average of the samples nearest each centroid, updating the cluster centroids to be equal to the average of the nearest samples, and iteratively repeating the process up to the point that the cluster centroid stops moving at each iteration above a pre-defined threshold. The main advantage of k-means clustering for use with dFNC is that it is easy to implement using existing libraries (45). However, while k-means clustering is widely used, it does have some disadvantages. First, it can yield low quality clusters. Previous studies have proposed approaches to try to address this problem (14). Additionally, it does not assign each sample a probability of belonging to each cluster centroid or resting-state, which involves an implicit assumption in subsequent analysis that the identified states are able to adequately summarize the dFNC time-series (46). This is a big assumption given that samples very near one another in the data space can be assigned to completely different states. In addition, in the hard clustering approach it can be hard to compare individuals as it is likely that some individuals may never enter a given state strongly enough to enter the cluster. As such, a simple fuzzy clustering-based approach could go a long way towards helping future studies in the field of dFNC clustering to uncover new aspects of disorder-related brain dynamics by better summarizing brain activity.

As the field of rs-fMRI FNC clustering has developed, a few studies have introduced explainability approaches with the goal of helping characterize the differences between identified states (7, 23, 47). In contrast to earlier efforts involving statistical hypothesis testing (38), these methods quantify the importance of FNC features in a manner that acknowledges the multi-dimensional nature of the underlying clustering. However, these methods have an important shortcoming that arises from their use with hard clustering methods. Specifically, they perturb FNC features and examine the sensitivity of the underlying clusters to perturbation. They quantify the effect of this perturbation upon the clustering by calculating the percentage of samples that completely switch clusters. Given that only a small minority of samples switch clusters following perturbation, it is safer to say that the methods quantify the effects of perturbation upon that subset of samples rather than upon the overall clustering. Similar to how a fuzzy clustering approach could help future studies better summarize brain activity and uncover new aspects of disorders, fuzzy clustering could also contribute to the development of explainability approaches that are better able to quantify the effects of perturbation.

In this study, we present fuzzy c-means clustering (48) as an approach for the identification of fMRI dFNC fuzzy states. Fuzzy c-means has been used widely across application areas like emotion recognition from speech data (49), customer segmentation (50), and brain image segmentation (51). Furthermore, fuzzy c-means is simple to use given its inclusion in several publicly available Python packages (52) and MATLAB (53). As such, it has the potential to be widely used within the domain of FNC clustering. Our approach outputs a probability that each sample belongs to each fuzzy state. After performing clustering, we present a pair of new explainability approaches to characterize the identified states, comparing them to an existing approach (47). We then present a variety of novel dynamical and stability features that highlight different aspects of the fuzzy state time-series while also showing that our approach is compatible with dynamical features that have been used in previous hard clustering studies. We further show how the features can be used to differentiate between healthy controls and individuals with schizophrenia in the default mode network (DMN), a network that has previously been associated with SZ (10, 12, 13, 27–32), and identify relationships between the features and SZ and SZ symptom severity.

## 2 Methods

**Figure 1:**
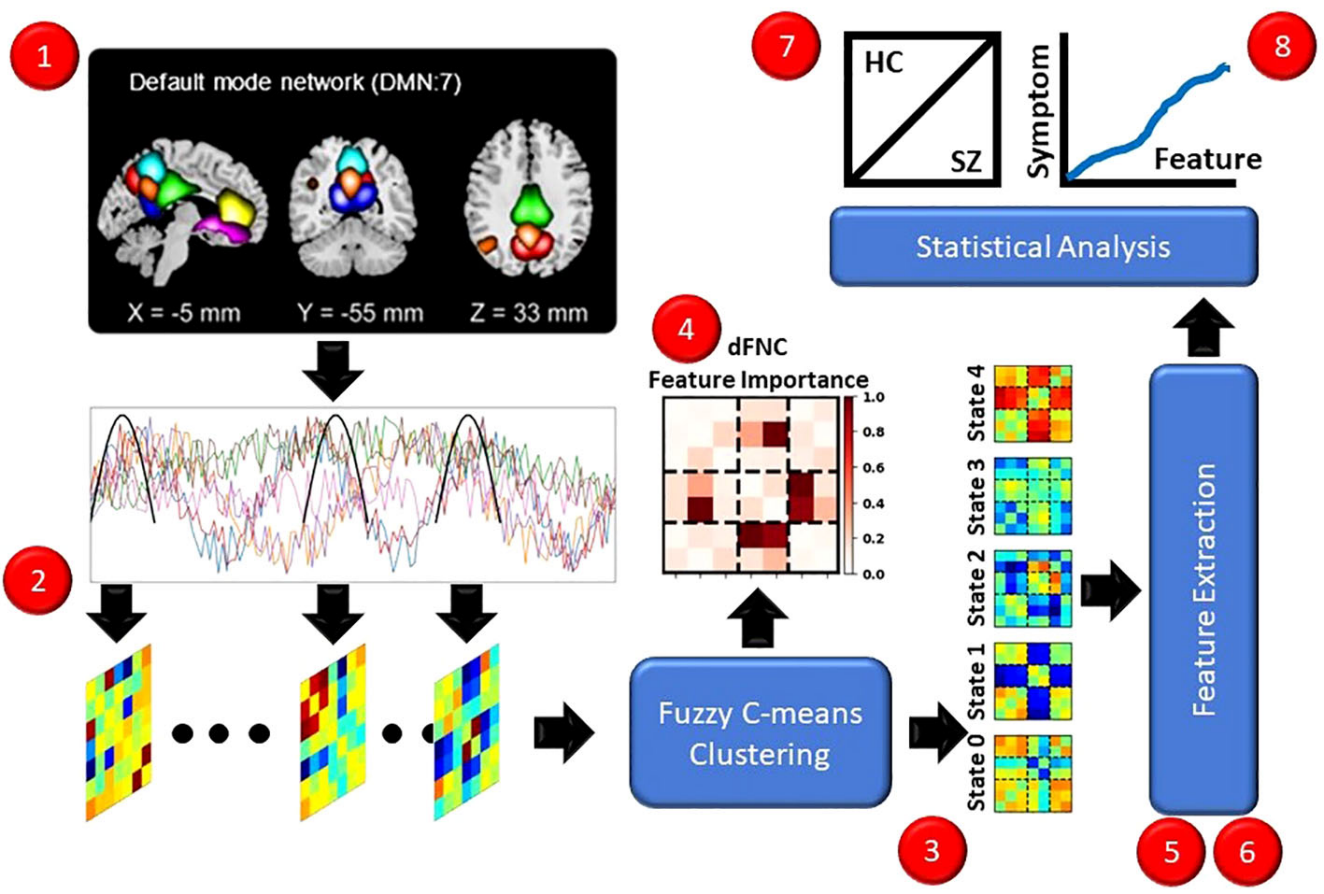
Overview of Methods. Red dots indicate each step of the methods. (1) We recorded rs-fMRI data from HCs and SZs. (2) We extracted dFNC data. (3) We performed fuzzy c-means clustering, identifying 5 states. (4) We applied several explainability approaches for insight into the dFNC features characterizing the states. (5, 6) We extracted dynamical and stability features. (7) We performed t-tests and trained interpretable machine learning models for insight into the features that differed between HCs and SZs. (8) We performed linear regression analyses controlling for age and gender to identify relationships between symptom severity and dynamical and stability features.

In this section, we describe and discuss our approach for the study. **Figure 1** presents an overview of our approach (1). We used a pre-existing rs-fMRI dataset composed of individuals with schizophrenia (SZs) and healthy controls (HCs). (2) We preprocessed the data and extracted dFNC. (3) We applied fuzzy c-means clustering to identify 5 fuzzy states. (4) We sought to identify the important dFNC features for each state by applying and comparing the results for two global clustering explainability methods. (5) We next extracted a number of pre-existing and novel dynamical features to summarize different aspects of the identified states. (6) We applied a novel local cluster explainability approach for insight into the stability of study participants to the perturbation of specific dFNC features. (7) To identify SZ-related differences in the extracted dynamical and stability features, we conducted statistical analyses and trained a series of logistic regression classifiers with elastic net regularization (LR-ENR). (8) Lastly, we performed statistical analyses to determine whether the dynamical and stability features were related to symptom severity. For the sake of reproducibility, our code can be found at: https://github.com/cae67/Fuzzy_Clustering/.

### 2.1 Description of dataset

In this study, we used the Functional Imaging Biomedical Informatics Research Network (FBIRN) dataset, consisting of rs-fMRI recordings from 151 SZs and 160 HCs (54). Participant demographics are shown in **Table 1**. The dataset has been used in many studies, both related to fMRI dFNC clustering and classification (21, 24, 43, 47). In addition to neuroimaging data, positive and negative symptom severity scores from the Positive and Negative Syndrome Scale (PANSS) (55). Positive symptoms of SZ include hallucinations, delusions, and bizarre behavior. Negative symptoms include alogia, apathy, affective flattening, and asociality (28). The dataset was collected at 7 sites: the University of California at Los Angeles, the University of California at San Francisco, the University of California at Irvine, the University of Iowa, the University of Minnesota, the University of New Mexico, and Duke University/the University of North Carolina at Chapel Hill. Data collection procedures were approved by the institutional review boards of each center, and all study participants gave written informed consent. One site used a 3T GE MR750 scanner, and 6 sites used 3T Siemens TIM Trio Scanners. Data was collected using a T2*-weighted AC-PC aligned EPI sequence (TE = 30ms, TR = 2s, slice gap = 1mm, flip angle = 77°, voxel size = 3.4 x 3.4 x 3.4 mm^3^, acquisition time = 5 min and 38s, and number of frames = 162).

**Table 1:** Description of Study Participants.

	Number	Age	Gender (M/F)	PANSS (positive)	PANSS (negative)
**SZ**	151	38.06 ± 11.30	115/36	15.32 ± 04.92	14.32 ± 05.42
**HC**	160	37.04 ± 10.68	115/45	not applicable	not applicable

### 2.2 Description of data preprocessing

Prior to preprocessing, the first 5 mock scans were removed. Statistical parametric mapping (SPM12, https://www.fil.ion.ucl.ac.uk/spm/) was used for preprocessing, and head motion was corrected using rigid body motion correction. The data was spatially normalized to an echo-planar imaging template in the standard Montreal Neurological Institute (MNI) space. Following resampling to 3x3x3 mm^3^, a Gaussian kernel with a 6mm full width at half maximum was used to smooth the data. The fully automated Neuromark Pipeline of the Group ICA of fMRI Toolbox (GIFT, http://trendscenter.org/software/gift) involved the use of spatially constrained ICA to extract corresponding components while adaptive to individual datasets. In our case, we used the neuromark_fMRI_1.0 template to extract 53 independent components (ICs) with peak activations in the gray matter of various brain networks. Seven of the ICs were associated with the DMN, which has been associated with SZ in multiple studies (10, 13, 28), and we used those components in this study. The 7 ICs included 3 precuneus (PCN) (56), 2 anterior cingulate cortex (ACC) (57), and 2 posterior cingulate cortex (PCC) (58). After IC extraction, dFNC was estimated using Pearson’s correlation with a sliding tapered window. The window consisted of a rectangle with a 40-second step size convolved with a Gaussian (σ=3). The window size parameter plays an important role in study outcomes. A shorter window size increases dynamics but can increase noise and susceptibility to artifacts, while longer window sizes decrease dynamics and are more robust to artifacts (59). While a number of window sizes have been utilized in previous studies, a 40-second size is highly common (13, 23, 37, 60). Additionally, a Gaussian with σ=3 is also highly common (3, 13, 36, 37). The dFNC extraction resulted in a dataset composed of 21 dFNC features and 124 time steps per participant. For the remainder of this paper, correlation between two brain regions is abbreviated using the pattern of IC1/IC2 (e.g., PCC1/ACC2), where IC1/IC2 is equivalent to IC2/IC1.

### 2.3 Description of clustering approach

After extracting dFNC, we concatenated all dFNC time steps across samples and applied fuzzy c-means clustering (48). Fuzzy c-means clustering is comparable to k-means clustering, but unlike k-means clustering, fuzzy c-means assigns each sample a probability of belonging to a given cluster. We used the Python package scikit-fuzzy in our implementation (52) and optimized the random seed used for initialization and the fuzziness parameter, m, based on the fuzzy partition coefficient (61). It is relatively common to set the number of clusters to a number identified in previous studies (62). As such, we used 5 clusters to more easily relate our findings to previous studies (13, 62) of SZ dFNC data. Further details on our cluster parameter optimization can be found in the **Supplementary Materials**.

### 2.4 Description of explainability approaches

We used three global explainability approaches for insight into the relative importance of each dFNC feature to the identified fuzzy states.

#### 2.4.1 Global permutation percent change feature importance

We applied Global Permutation Percent Change (G2PC) feature importance (47), which has been used in several neuroimaging studies (7, 23). G2PC extends permutation feature importance (63, 64) to the clustering domain, wherein individual features are permuted and the features that cause the greatest percentage of samples that switch clusters are considered most important. Further details on G2PC can be found in the **Supplementary Materials**.

#### 2.4.2 Permutation-based distribution divergence and G2PC comparison

We used two variations of a novel approach called, “Permutation-based Distribution Divergence (P2D)”. P2D has global (GP2D) and local (LP2D) variations (65). P2D is similar to G2PC. However, rather than computing the percent of samples that switch clusters after permutation, P2D involves calculating the Kullback-Leibler divergence (KLD) between the probabilities of a sample belonging to each cluster before versus after permutation. For GP2D, the median and total KLD across samples were calculated, and the rankings of GP2D feature importance were compared with the G2PC feature importance rankings (66–68). For our analysis with LP2D, we (1) compared the percentage of samples with non-zero KLD values with the G2PC results to see if P2D was more sensitive than G2PC and (2) compared HC and SZ KLD values to identify differences in cluster stability. Further details on our P2D and comparison analyses can be found in the **Supplementary Materials**.

### 2.5 Description of dynamical feature extraction

After clustering all of the samples, we extracted features to quantify different aspects of the dynamics of the transition to and from the identified fuzzy states. We used two types of features that have been frequently used in hard cluster-based studies (1, 5, 36, 37) and developed a number of new features uniquely suited for use with fuzzy clustering that give new insight into state dynamics. For traditional hard cluster-based features, we assigned each sample to the cluster for which it had the highest probability and extracted the occupancy rate (OCR, i.e., the percent of time steps spent in a state by a participant) and number of state transitions (NST). For novel fuzzy cluster-based features, we extracted the (1) Kullback-Leibler divergence (KLD) across states and calculated a variety of descriptive statistics summarizing the KLD (5 features total). (2) We also calculated Shannon entropy over time for each fuzzy state (1 feature per state, so 5 features total). (3) We calculated 3 descriptive statistics for probabilities for each state (15 features total). We calculated (4) the cumulative change between consecutive time points for each state (5 features total) and (5) a measure of the uniformity of probabilities within each state (5 features total). We sought to use these features to understand the effects of SZ upon the DMN dynamics, and statistical analyses using the features will be described in subsequent sections. Further details on each feature can be found in the **Supplementary Materials**.

### 2.6 Description of class-related statistical analysis

We wanted to determine whether the fuzzy states that we identified were related to SZ dynamics. As such, we performed a series of two-tailed t-tests comparing the dynamical and LP2D stability features belonging to SZs to those belonging to HCs. After performing the t-tests, we performed separate FDR corrections on the OCR features, correlation-based features, cumulative change features, and LP2D-based features to reduce the likelihood of false positive test results.

### 2.7 Description of LR-ENR classification-based explainability analysis

Our statistical analysis indicated whether there were significant differences in the extracted dynamical features between HCs and SZs and gave insight into the effects of SZ upon DMN dynamics. However, we also wanted to determine how helpful the extracted features could be for discriminating between HCs and SZs and gain insight into the relative importance of each feature for the classification. As such, we classified SZs and HCs using LR-ENR. LR-ENR is a frequently used (13) interpretable machine learning model. Its coefficients can be visualized for insight into the relative importance of each feature included in the classification. In our Scikit-Learn implementation (45), we feature-wise z-scored each feature and then trained separate LR-ENR classifiers for the traditional, non-uniformity + KLD + entropy, variance, mean, range, correlation, cumulative difference, and LP2D features. We used 10-fold nested cross-validation to optimize the ratio of L1 to L2 normalization and the regularization strength. We lastly calculated the mean and standard deviation of the area-under-curve (AUC) of the receiver-operating-curve, sensitivity (SENS, i.e., true positive rate), and specificity (SPEC, i.e., true negative rate) for the 10 folds corresponding to each set of dynamical features. Details on our parameter optimization approach can be found in the **Supplementary Materials**.

### 2.8 Description of symptom severity analysis

Lastly, while our earlier analyses gave insight into differences in DMN dynamics between SZs and HCs, we also wanted to determine whether the fuzzy states we uncovered could be used for insight into SZ symptom severity. As such, we performed ordinary least squares regression using age, gender, one-hot encoded site data and the negative PANSS score or age, gender, one-hot encoded site data, and the positive PANSS score as independent variables and the dynamical features as independent variables. We lastly applied FDR correction for the OCR features, entropy features, correlation-based features, cumulative change features, and LP2D-based features separately.

## 3 Results

In this section, we describe the 5 fuzzy states that we identified with our clustering approach. We then show the important dFNC features identified using our global explainability analyses. After characterizing the states and the dFNC features that differentiate them, we examine whether there are differences in the dynamical features and LP2D-based stability features between HCs and SZs and whether the features are related to symptom severity.

### 3.1 Identifying 5 fuzzy states of dFNC activity

**Figure 2:**
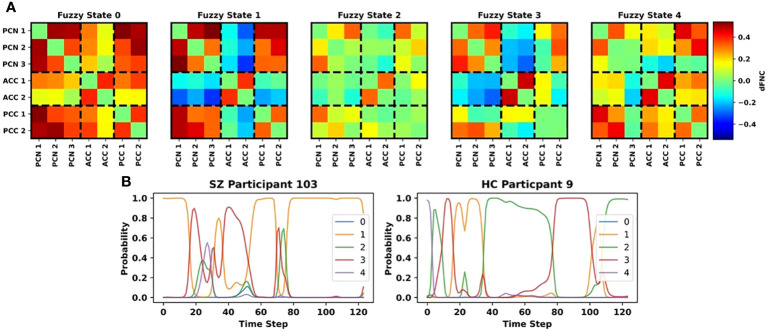
Fuzzy States and Example State Trajectories. **(A)** shows the centroids for each of the 5 fuzzy states that we identified. Each subpanel shares the same color bar to the right of the panel for Fuzzy State 4. The ICs associated with each dFNC feature are arranged on the x- and y-axes and are grouped based upon brain region (i.e., PCN, ACC, PCN). **(B)** shows example state trajectories for an SZ participant (left) and HC participant (right). The y-axis indicates the probability of belonging to each state, and the different colors of lines correspond to the state numbers shown in the legends. Note the variation in probability of belonging to each cluster.

As shown in **Figure 2**, we identified 5 distinct fuzzy states of dFNC activity. All states had highly positive intra-ACC dFNC. State 0 had overall highly positive dFNC values. It had moderately highly levels of positive ACC/PCN and ACC2/PCC dFNC. State 1 was characterized as having highly negative ACC/PCN and ACC/PCC dFNC, highly positive intra-network dFNC, and highly positive PCC/PCN dFNC. Relative to the other states, state 1 had more higher magnitude patterns of positive and negative dFNC. State 2 was the most poorly connected state, with relatively low values of positive dFNC. However, PCN1/PCN3, PCN1/PCC1, and intra-ACC had moderate positive values. Similar to state 1, state 3 had highly predominantly negative, though lower magnitude, ACC/PCN dFNC. State 3 also had higher magnitude positive intra-PCN activity relative to states 2 and 4. It had very low intra-PCC and PCC/ACC dFNC comparable to state 2. Additionally, PCC/PCN and particularly PCC1/PCN were lower magnitude than every state but state 2. Lastly, state 4 had lower magnitude intra-PCN and ACC/PCN dFNC comparable to state 2. It had levels of intra-ACC and intra-PCC dFNC comparable to states 0 and 1. It had moderately positive PCC/ACC dFNC comparable to state 0 and positive PCC/PCN dFNC comparable to states 0 and 1.

### 3.2 Identifying key dFNC features differentiating each state and comparing explainability approaches

**Figure 3:**
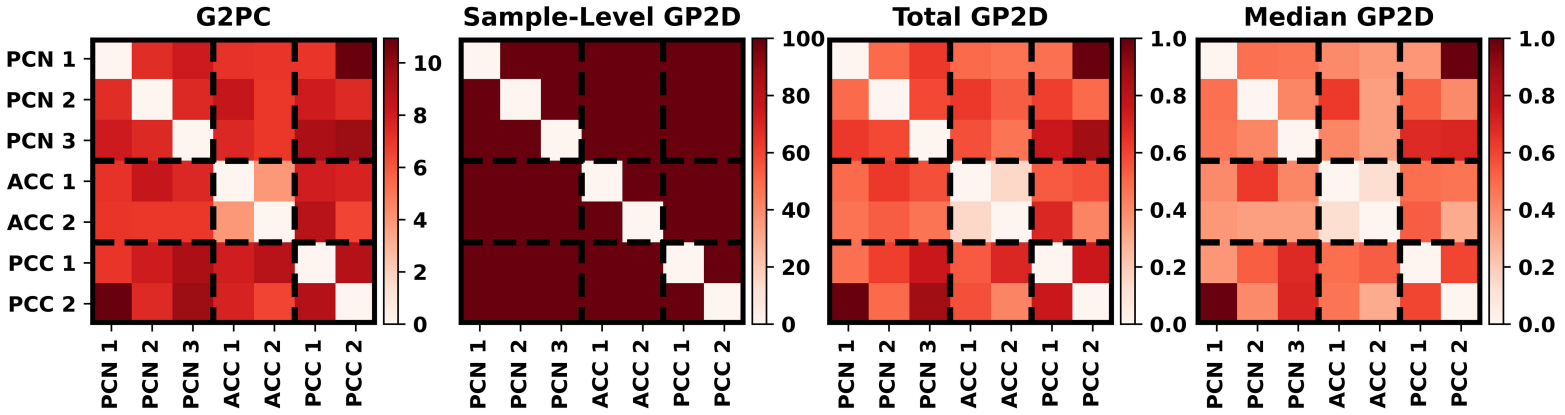
Explainability Results. From left to right, the panels show the G2PC, LP2D, total GP2D, and median GP2D results. The color bar corresponding to each panel is positioned to its right, and the ICs associated with each dFNC feature are arranged on the x- and y-axes and are grouped based upon brain region (i.e., PCN, ACC, PCN). Total GP2D and median GP2D are scaled by their maximal value for easier interpretation.

Although we visually compared the centroids of the clusters that we identified, the visual comparison did not address the relative importance of each dFNC feature to the identified states in a quantitative manner and did not consider the underlying distribution of the samples in relation to the identified fuzzy states. **Figure 3** shows the results for our global explainability analyses. G2PC, total GP2D, and median GP2D all identified PCC2/PCN1 and PCC/PCN3 to be among the most important dFNC features with the other features being of varying importance. G2PC affected around 10% of samples. In contrast, mean LP2D captured the effects of perturbation on 100% of samples. Additionally, based on Kendall’s rank correlation between the global feature importance of each method, there was significant high-level agreement in the relative importance (i.e., importance rank) of each dFNC feature across global approaches (e.g., the most important features for one method tended to be among the most important for the other methods). G2PC and total GP2D (p < 0.001) had a correlation coefficient of 0.65. G2PC and median GP2D (p < 0.001) had a correlation coefficient of 0.53, and total GP2D and median GP2D (p < 0.05) had a correlation coefficient of 0.39. Additionally, while G2PC and total GP2D tended to distribute importance widely, median GP2D provided a much sparser importance estimate.

### 3.3 Identifying disorder-related differences in dynamical and stability features

**Figure 4:**
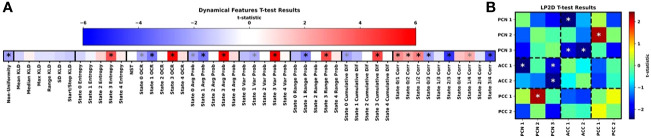
Group-Level Statistical Comparison. **(A, B)** show the t-test results for the dynamical and LP2D stability features, respectively. In **(A)**, the groups of features we extracted are arranged from left to right, and dark black lines separate each group of features. In **(B)**, results are arranged in the form of a standard connectivity matrix for easier interpretation. Dashed lines separate domain pairs. Both panels are heatmaps showing the t-statistics for each feature. The color bar for **(A)** is above the heatmap, and the color bar for **(B)** is to the right of the heatmap. Black and grey asterisks indicate features with significant differences with and without FDR correction, respectively It should be noted that the t-tests were performed as HCs minus SZs. As such, a negative t-statistic indicates that SZs had higher values for a particular feature than HCs.

**Figure 4** shows the results for our t-tests examining differences in dynamical and LP2D stability features between HCs and SZs, and **Supplementary Figures 1-4** show boxplots of the dynamical features for each class. Many features, though not the KLD features, had statistically significant differences between groups. State 3 was of reoccurring importance, as HCs had significantly higher entropy, OCR, average probabilities, range in probabilities, and cumulative difference values in state 3. Interestingly, SZs had greater non-uniformity in their distribution of probabilities across states and greater state 0 and state 1 OCRs and average probabilities. SZs also had greater state 1 range in probabilities and state 0 cumulative differences. Additionally, many state correlations displayed significant differences between groups. HCs had higher state 0/1, 0/2, 1/2, and 1/4 correlations, and SZs had higher state 0/3 and 2/3 correlations. Lastly, SZs had higher levels of sensitivity to ACC1/PCN1, ACC1/PCN3, and ACC2/PCN3 perturbation than HCs and lower levels of sensitivity to PCC1/PCN2 perturbation than HCs.

**Figure 5:**
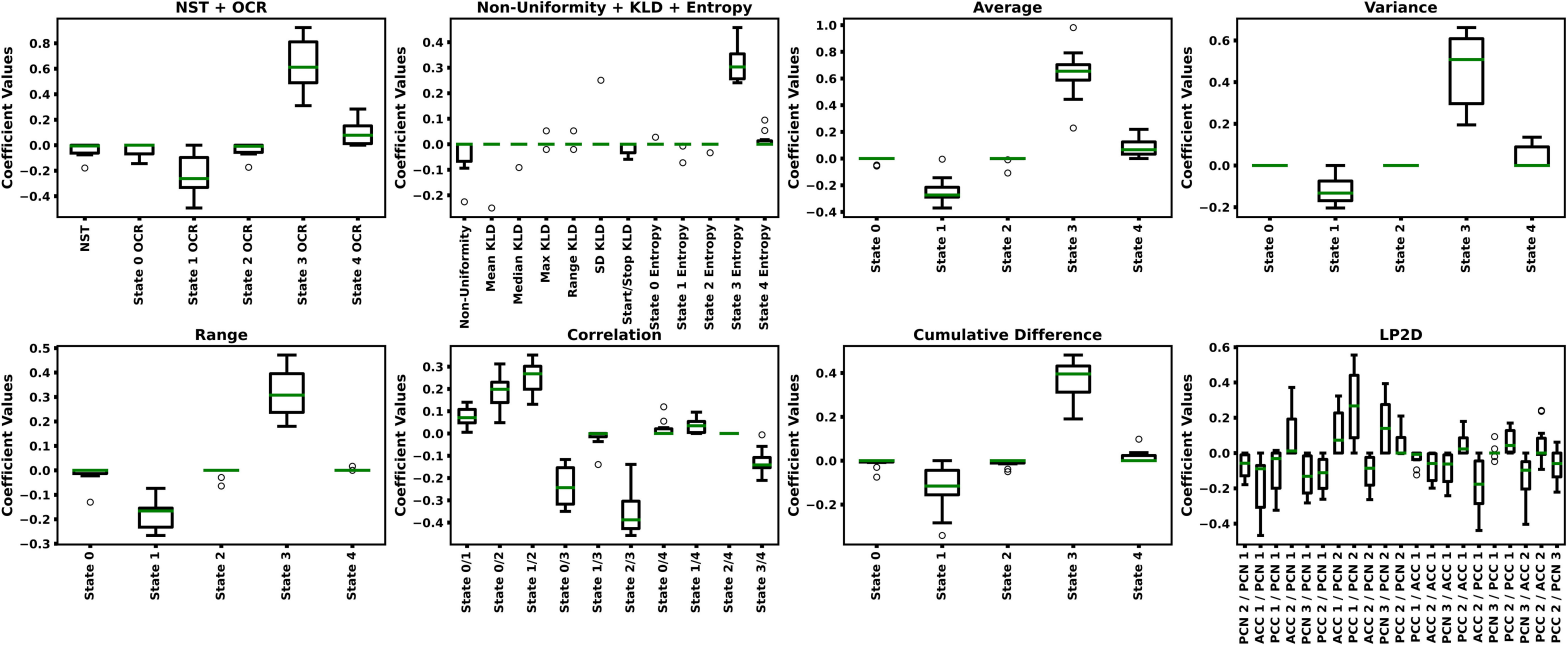
LR-ENR Explainability Results. Each panels shows the LR-ENR coefficient values for the groups of features indicated in their respective titles. Specific feature names are indicated on the x-axis of each panel. Coefficient values are shown on the y-axis. Higher magnitude coefficients indicate greater importance to their respective model. Because a label of 0 was used for SZs and a label of 1 was used for HCs, a negative coefficient value indicates that an increase in that feature corresponded to an increase in likelihood of belonging to the SZ class.

**Table 2** shows the LR-ENR performance results, and **Figure 5** shows the LR-ENR explainability results. As might be expected, given the t-test results, the Correlation, Average, Variance, NST + OCR, and Range feature models had the highest AUC values. The LP2D and Non-Uniformity + KLD + Entropy feature models had near chance-level performance. The Correlation model had the highest overall AUC. For SENS, the NST + OCR and Average feature models performed highest, with Variance feature model performing slightly lower. For SPEC, the Range, and Correlation, and LP2D models performed highest. The model coefficients also provided results highly consistent with the t-test results, exhibiting similar directionality and relative magnitude of differences between HCs and SZs.

**Table 2:** LR-ENR Performance Results.

Features	AUC	SENS	SPEC
**NST + OCR**	66.42 ± 06.88	64.52 ± 05.95	55.62 ± 08.93
**Non-Uniformity + KLD + Entropy**	58.76 ± 04.68	51.94 ± 07.56	59.06 ± 07.96
**Average**	68.12 ± 06.76	64.52 ± 06.12	59.69 ± 07.58
**Variance**	66.88 ± 06.53	63.55 ± 07.50	59.69 ± 07.05
**Range**	65.24 ± 06.39	43.87 ± 07.10	76.56 ± 08.18
**Correlation**	68.31 ± 04.48	59.03 ± 05.41	65.94 ± 06.32
**Cumulative Difference**	62.64 ± 08.64	56.45 ± 09.81	35.48 ± 15.34
**LP2D**	49.47 ± 03.57	35.48 ± 15.34	65.94 ± 15.96

### 3.4 Identifying relationship between symptom severity and dynamical and stability features

Interestingly, no stability features had significant relationships with or without FDR correction with symptom severity when accounting for age, gender, and study site, so we did not include a figure detailing the results for that analysis. However, accounting for age, gender, and study site, state 2 cumulative difference was positively correlated with the negative PANSS score (p < 0.05) prior to FDR correction.

## 4 Discussion

The goals of this study were (1) to present a framework for dFNC state analysis that accounts for the inherent variability of data within states and (2) provide additional insights into the effects of SZ upon DMN dynamics. We identified 5 fuzzy states of DMN activity, characterized the states using a novel explainability approach, identified effects of SZ upon DMN dynamics and state stability, and identified relationships between DMN dynamics and symptom severity.

Interestingly, the centroids for the 5 fuzzy states that we identified diverge greatly from those identified in previous studies (13). Nevertheless, there were some overall similarities in dFNC (13). Multiple states in (13) had highly positive intra-PCN dFNC and highly positive PCN/PCC dFNC. In contrast to the clusters that we identified, most of the centroids in (13) had negative intra-ACC and intra-PCC dFNC, while our clusters had positive values. Additionally, dFNC values seemed to be much more uniform in our clusters, whereas those in (13) had much more variability of dFNC values between independent components in each state.

While not all of our dynamic and stability features were able to obtain LR-ENR performance, comparable to the features extracted in (13), mean AUC for the NST + OCR, correlation, average probability, and variance features were comparable, which supports the utility of our pipeline for identifying key class-specific differences in brain dynamics. Importantly, HCs spent much more time in and had higher average probabilities for a state characterized by moderately negative ACC/PCN, moderately positive intra-PCN, and low magnitude intra-PCC and ACC/PCC dFNC (state 3). As might be expected given that they spent more time within that state, HCs also had much higher variability within that state (i.e., higher levels of variance, entropy, range in probabilities, and cumulative difference) than SZs in the state. In contrast, SZs spent more time in a state with highly negative ACC/PCN, moderately negative ACC/PCC dFNC, highly positive intra-network dFNC, and highly positive PCC/PCN dFNC (state 1). ACC/PCC activity was highly important to SZ dynamics, as they lacked stability to perturbation of ACC/PCC activity. Our findings of more negative or less positive ACC/PCC dFNC in SZ fit with those of multiple studies. Many studies have identified reduced or disrupted ACC/PCC dFNC in SZs (13, 30, 69, 70), and these differences could be related to reduced ACC and PCC grey matter volume in SZs (71) or the disrupted anatomical distance function found in SZ (72). State 1 also had higher intra-PCN activity than state 3, which corroborates the findings of previous studies that identified higher intra-PCN activity in SZs than HCs (31, 73). Previous studies have also suggested that this overactivation of the PCN could be a compensatory mechanism to reduce language comprehension deficits found in SZ (73) or that the PCN in SZ could be related to differentiating fantasy from reality (74) and that overactivation tends to be related to increased symptom severity. SZs also tended to be less stable to ACC/PCN perturbation, whereas HCs tended to be less stable than SZs to PCC/PCN perturbation. These findings paired with a visual comparison of state centroids indicates that strongly negative ACC/PCN activity and strongly positive PCC/PCN activity were important for identifying SZs. Previous studies have found that the PCN strongly inhibits the ACC, which corroborates our finding of strongly negative ACC/PCN activity (75). Although many DMN studies agree with our finding that SZs spend more time in a state with more positive connectivity (31, 76) (i.e., PCN/PCN, PCC/PCN, and PCC/PCC in state 1 versus state 3), many previous studies have found SZ inter-domain connectivity to be less than HCs (10). This difference in results is likely attributable to the use of different regions within the DMN (10, 77), the use of whole-brain analyses (77) that have been shown to obscure the activity of the DMN (13, 23), or the use of multiple disorders that can disguise the effects of SZ (12).

Though there are key differences, the insights that can be obtained from our state correlation features are somewhat related to the hidden Markov model state transition probabilities from (13). While the features in (13) indicate the probability of transitioning between a given state, our correlations features indicate how the similarity of brain dynamics of study participants to each fuzzy state vary over time and relate to multiple states simultaneously. SZs spent more time in states 0 and 1, which had high-magnitude dFNC with the exception of negative ACC/PCC and ACC/PCN in state 1. Some findings related to our correlation features provide helpful insight into SZ, though others are likely explained by aspects of our results that were previously explained. Importantly, similarity to state 0 and state 1 activity tended to be more strongly anticorrelated in SZs, and given that SZs spend a significant amount of time in each of those states, that would indicate that there was a large amount of alternation between the states (i.e., changes in ACC/PCC and ACC/PCN activity). Given that State 2 (low magnitude dFNC) was the dominant state, stronger anticorrelations between states 0/2 and states 1/2 in SZs could be explained by their stronger similarity to states 0 and 1 (i.e., being closer to a state of high magnitude dFNC is being farther from a state of low magnitude dFNC) or by increased transitions between states of high magnitude dFNC (i.e., states 0 and 1) and a state of low magnitude dFNC (state 2). Additionally, states 1 and 4 in SZs had stronger anticorrelation than in HCs, which could be explained by the greater state 1 occupancy of SZs, and SZs had more positive correlation between states 2 and 3, which could be explained by their relatively low similarity to each state 3. Our findings of stronger anticorrelation between states 0 and 3 and states 2 and 3 in HCs could also be explained to indicate that HCs transitioned more between states of moderate and high magnitude dFNC or states of low and moderate dFNC. Together, these findings could suggest that SZs transition more rapidly from low-magnitude dFNC states to high-magnitude states, while HCs transition more gradually from low-magnitude to moderate-magnitude to high-magnitude states. This could corroborate findings of previous studies that have identified the effects of SZ to involve more temporally localized changes in activity (22–24).

It is unfortunate that our KLD analysis did not uncover any significant differences between SZs and HCs. Previous studies have uncovered significant effects of SZ upon dynamics. However, our lack of findings related to KLD features does not indicate that those features would not provide useful insights within the context of other applications. While we extracted a number of metrics to quantify different aspects of the KLD, it is also possible that other metrics might be applied to obtain other insights into state dynamics.

Several studies have previously identified relationships between DMN dFNC features and symptom severity (12, 13). We found that our use of the cumulative difference of fuzzy clustering probabilities uncovered a key relationship with negative symptom severity. Accounting for age, gender, and study site, the state 2 cumulative difference was related to negative symptom severity. This indicates that the more variation SZs had in their similarity to a state of low-magnitude dFNC, the worse their symptoms may have been. Given that previous studies have related DMN deactivation with SZ symptom severity (78–80), it is necessary to be careful with this conclusion as the result was only found prior to FDR correction.

While our findings hold great significance for the domain of SZ analysis, our proposed explainable fuzzy clustering framework has broader implications. Our use of fuzzy clustering enables insight not only into inter-state dynamics but also into intra-state dynamics. Furthermore, our approach accounts for the inherent variation in how similar samples are to their assigned centroid and to samples assigned to other centroids. These challenges will be present in any dFNC clustering analysis. Our dynamical and stability features also represent key advances, providing new insights into disorder-related activity. Additionally, as we showed by thresholding the state probabilities and calculating OCR and NST values, traditional k-means-based features can still be extracted with fuzzy c-means. That paired with the ease of implementation of fuzzy c-means using existing Python packages (52) and MATLAB (53) means that our approach could easily replace existing dFNC k-means clustering approaches in future studies. The explainability methods that we present also represent advances for the field, providing a quantitative estimate of the importance of each dFNC feature to the clustering. Relative to G2PC, our approach accounts for the effects of perturbation upon 100% of samples. Additionally, different metrics can be applied to the resulting KLD values to gain different insights into the effects of perturbation or to produce more or less sparse explanations (e.g., in total versus median KLD).

### 4.1 Limitations and future opportunities

Our study and approach have several limitations. Namely, we used a 40-second window when calculating dFNC, and while studies have shown that to be a reasonable window size (60), the window size can affect the dynamics and findings. Additionally, it is possible that our use of a feature extraction approach (clustering followed by extracting dynamical and stability features) applied to the whole dataset may have biased our LR-ENR performance results (81). However, our methods did enable us to directly compare the utility of our novel extracted features to those developed in (13). In future studies, we intend to perform a more robust series of analyses on how to best optimize the number of clusters for our application area. However, in this study, we used 5 clusters for easier comparison to (13). Fuzzy c-means has a couple limitations (1). Similar to k-means clustering, it is affected by outliers. However, there are variations of fuzzy c-means that are capable of addressing this problem (82). Additionally, fuzzy c-means can be more computationally intensive than k-means clustering (83). However, an approach similar to iSparse k-means could be easily adapted to work with fuzzy c-means (84). Lastly, future research directions might include analyzing the reproducibility of our results across datasets similar to (13) or analyzing multiple disorders in a single analysis like (4).

## 5 Conclusion

The analysis of rs-fMRI dFNC data using hard clustering methods to identify states that summarize brain dynamics is a common analysis approach that has provided insights into many neurological and neuropsychiatric disorders. However, the use of hard clustering approaches (e.g., k-means clustering) can obscure key information related to how similar samples are to their respective centroids or to samples assigned to other cluster centroids. As such, the use of hard clustering could obscure disorder-relevant dynamics. In this study, we present a novel explainable fuzzy clustering framework. We present 7 new types of dynamical features and sample stability-based features that provide unique insights into brain dynamics while also demonstrating how traditional dynamical features used in hard clustering analyses can also be included in our analysis. Lastly, we present two novel explainability approaches that help characterize the fuzzy states identified using our clustering approach. We demonstrate our framework within the context of SZ DMN analysis, identifying aberrant dynamics in SZs and uncovering relationships between SZ symptom severity and a state of reduced DMN correlation. Our framework provides greater insight into disease dynamics than traditional hard clustering approaches. Furthermore, it can be implemented with an ease comparable to the standard k-means clustering approach using existing code packages. As such, it represents an ideal method for future widespread use in rs-fMRI dFNC analysis and could lead to an improved understanding of the effects of many neurological and neuropsychological disorders upon brain dynamics.

## Data availability statement

The data analyzed in this study is subject to the following licenses/restrictions: The dataset analyzed in this study can be made available pending a reasonable request. Requests to access these datasets should be directed to Vince Calhoun, vcalhoun@gsu.edu.

## Ethics statement

The data involved in this study were obtained from the Mind Research Network Center of Biomedical Research Excellence (COBRE) and the FBIRN projects. The FBIRN raw imaging data were collected from seven sites including the University of California, Irvine. the University of California, Los Angeles, the University of California, San Francisco, Duke University/the University of North Carolina at Chapel Hill, the University of New Mexico, the University of Iowa, and the University of Minnesota. The studies were conducted in accordance with the local legislation and institutional requirements. The participants provided their written informed consent to participate in this study.

## Author contributions

CE helped with conception of the paper, performed analyses, wrote the paper, and edited the paper. RM helped with the conception and editing of the paper. VC helped with the editing of the paper and provided funding for the paper. All authors contributed to the article and approved the submitted version.

## References

[1] Sendi MSE Zendehrouh E Ellis CA Chen J Miller RL Mormino EC. The link between brain functional network connectivity and genetic risk of Alzheimer’s disease. NeuroImage Clin (2023) 37:103363. doi: 10.1002/alz.050101 36871405 PMC9999198

[2] Damaraju E Allen EA Belger A Ford JM McEwen S Mathalon DH. Dynamic functional connectivity analysis reveals transient states of dysconnectivity in schizophrenia. NeuroImage Clin (2014) 5:298–308. doi: 10.1016/j.nicl.2014.07.003 25161896 PMC4141977

[3] Zendehrouh E Sendi MSE Sui J Fu Z Zhi D Lv L. Aberrant functional network connectivity transition probability in major depressive disorder, in: 42nd Annual International Conference of the IEEE Engineering in Medicine and Biology Society (EMBC). Montreal, QC, Canada: IEEE. (2020) pp. 1493–6. 10.1109/EMBC44109.2020.9175872 PMC8233065 33018274

[4] Wu X LL Z Shen H Yuan L Qin J Zhang P. Functional network connectivity alterations in schizophrenia and depression. Psychiatry Res - Neuroimaging (2017) 263:113–20. doi: 10.1016/j.pscychresns.2017.03.012 28371656

[5] Dini H Sendi MSE Sui J Fu Z Espinoza R Narr KL. Dynamic functional connectivity predicts treatment response to electroconvulsive therapy in major depressive disorder. Front Hum Neurosci (2021) 15:689488. doi: 10.3389/fnhum.2021.689488 34295231 PMC8291148

[6] Chun JY Sendi MSE Sui J Zhi D Calhoun VD. Visualizing functional network connectivity difference between healthy control and major depressive disorder using an explainable machine-learning method, in: 2020 42nd Annual International Conference of the IEEE Engineering in Medicine & Biology Society (EMBC). Montreal, Canada: IEEE. (2020) pp. 955–60. doi: 10.1109/BIBE50027.2020.00162 33018257

[7] Ellis CA Sancho ML Miller R Calhoun V. Exploring relationships between functional network connectivity and cognition with an explainable clustering approach, in: 2022 IEEE 22nd International Conference on Bioinformatics and Bioengineering (BIBE). Taichung, Taiwan: IEEE. (2022) pp. 23–6. doi: 10.1109/BIBE55377.2022.00066

[8] Sendi MSE Ellis CA Milller RL Salat DH Calhoun VD. The relationship between dynamic functional network connectivity and spatial orientation in healthy young adults. bioRxiv (2021). doi: 10.1101/2021.02.14.431143

[9] Sanfratello L Houck J Calhoun VD. Dynamic functional network connectivity in schizophrenia with MEG and fMRI, do different time scales tell A different story? Brain Connect (2019), 251–62. doi: 10.1089/brain.2018.0608 PMC6479258 30632385

[10] Du Y Pearlson GD Yu Q He H Lin D Sui J. Interaction among subsystems within default mode network diminished in schizophrenia patients: A dynamic connectivity approach. Schizophr Res (2016) 170:55–65. doi: 10.1016/j.schres.2015.11.021 26654933 PMC4707124

[11] Miller RL Yaesoubi M Turner JA Mathalon D Preda A Pearlson G. Higher dimensional meta-state analysis reveals reduced resting fMRI connectivity dynamism in schizophrenia patients. PloS One (2016) 11:1–24. doi: 10.1371/journal.pone.0149849 PMC4794213 26981625

[12] Sendi MSE Dini H Bruni LE Calhoun VD. Default mode network dynamic functional network connectivity predicts psychotic symptom severity, in: Proceedings of the Annual International Conference of the IEEE Engineering in Medicine and Biology Society, EMBS. Glasgow, Scotland: IEEE. (2022) pp. 247–50. doi: 10.1109/EMBC48229.2022.9871542 36085610

[13] Sendi MSE Zendehrouh E Ellis CA Liang Z Fu Z Mathalon DH. Aberrant dynamic functional connectivity of default mode network in schizophrenia and links to symptom severity. Front Neural Circuits (2021) 15:649417. doi: 10.3389/fncir.2021.649417 33815070 PMC8013735

[14] Salman MS Du Y Calhoun VD. Identifying FMRI dynamic connectivity states using affinity propagation clustering method: Application to schizophrenia, in: ICASSP, IEEE Int Conf Acoust Speech Signal Process - Proc. New Orleans, USA: IEEE. (2017) pp. 904–8. doi: 10.1109/ICASSP.2017.7952287

[15] Abrol A Damaraju E Miller RL Stephen J Claus E Mayer A. Replicability of time-varying connectivity patterns in large resting state fMRI samples. Neuroimage (2017) 163:160–76. doi: 10.1016/j.neuroimage.2017.09.020.Replicability PMC5775892 28916181

[16] Ellis CA Miller RL Calhoun VD. A convolutional autoencoder-based explainable clustering approach for resting-state EEG analysis. 45th Annual International Conference of the IEEE Engineering in Medicine and Biology Society. Sydney, Australia: IEEE (2023), 1–4. doi: 10.1109/EMBC40787.2023.10340375 38083554

[17] Ellis CA Sattiraju A Miller R Calhoun V. Examining effects of schizophrenia on EEG with explainable deep learning models. 2022 IEEE 22nd Int Conf Bioinf Bioengineering (BIBE). (2022). doi: 10.1109/BIBE55377.2022.00068

[18] Ellis CA Sattiraju A Miller R Calhoun V. Examining reproducibility of EEG schizophrenia biomarkers across explainable machine learning models, in: 2022 IEEE 22nd International Conference on Bioinformatics and Bioengineering (BIBE). Taichung, Taiwan: IEEE. (2022) pp. 305–8. doi: 10.1109/BIBE55377.2022.00069

[19] Gawne TJ Overbeek GJ Killen JF Reid MA Kraguljac NV Denney TS. A multimodal magnetoencephalography 7 T fMRI and 7 T proton MR spectroscopy study in first episode psychosis. NPJ Schizophr (2020) 6:1–9. doi: 10.1038/s41537-020-00113-4 32887887 PMC7473853

[20] Yan W Calhoun V Song M Cui Y Yan H Liu S. Discriminating schizophrenia using recurrent neural network applied on time courses of multi-site FMRI data. EBioMedicine (2019) 47:543–52. doi: 10.1016/j.ebiom.2019.08.023 PMC6796503 31420302

[21] Ellis CA Miller RL Calhoun VD. An Approach for Estimating Explanation Uncertainty in fMRI dFNC Classification. 2022 IEEE 22nd Int Conf Bioinforma Bioeng (2022). doi: 10.1109/BIBE55377.2022.00067

[22] Ellis CA Miller RL Calhoun VD. Towards greater neuroimaging classification transparency via the integration of explainability methods and confidence estimation approaches. Inf Med Unlocked (2023) 37. doi: 10.1016/j.imu.2023.101176 PMC10078989 37035832

[23] Ellis CA Sendi MSE Miller RL Calhoun VD. An unsupervised feature learning approach for elucidating hidden dynamics in rs-fMRI functional network connectivity, in: 2022 44th Annual International Conference of the IEEE Engineering in Medicine & Biology Society (EMBC). Glasgow, Scotland: IEEE. (2022) pp. 4449–52. 10.1109/EMBC48229.2022.9871548 36086408

[24] Rahman M Lewis N Fedorov A Calhoun V Rahman M Mahmood U. Interpreting models interpreting brain dynamics. Sci Rep (2022) 12:1–16. doi: 10.1038/s41598-022-15539-2 35864279 PMC9304350

[25] Bliksted V Frith C Videbech P Fagerlund B Emborg C Simonsen A. Hyper- and hypomentalizing in patients with first-episode schizophrenia: FMRI and behavioral studies. Schizophr Bull (2019) 45:377–85. doi: 10.1093/schbul/sby027 PMC6403062 29534245

[26] Ebisch SJH Gallese V Salone A Martinotti G di Iorio G Mantini D. Disrupted relationship between “resting state” connectivity and task-evoked activity during social perception in schizophrenia. Schizophr Res (2018) 193:370–6. doi: 10.1016/j.schres.2017.07.020 28735643

[27] Shukla DK Wijtenburg SA Chen H Chiappelli JJ Kochunov P Hong LE. Anterior cingulate glutamate and GABA associations on functional connectivity in schizophrenia. Schizophr Bull (2019) 45:647–58. doi: 10.1093/schbul/sby075 PMC6483591 29912445

[28] Hare SM Ford JM Mathalon DH Damaraju E Bustillo J Belger A. Salience-default mode functional network connectivity linked to positive and negative symptoms of schizophrenia. Schizophr Bull (2019) 45:892–901. doi: 10.1093/schbul/sby112 30169884 PMC6581131

[29] Kottaram A Johnston LA Cocchi L Ganella EP Everall I Pantelis C. Brain network dynamics in schizophrenia: Reduced dynamism of the default mode network. Hum Brain Mapp (2019) 40:2212–28. doi: 10.1002/hbm.24519 PMC6917018 30664285

[30] Chand GB Thakuri DS Soni B. Kingshighway Blvd St Louis S. Disrupted controlling mechanism of salience network on default-mode network and central-executive network in schizophrenia. bioRxiv (2021), 1–19. doi: 10.1101/2021.12.03.471183

[31] Whitfield-Gabrieli S Thermenos HW Milanovic S Tsuang MT Faraone SV McCarley RW. Hyperactivity and hyperconnectivity of the default network in schizophrenia and in first-degree relatives of persons with schizophrenia. Proc Natl Acad Sci USA (2009) 106:1279–84. doi: 10.1073/pnas.0809141106 PMC2633557 19164577

[32] Yu Q Calhoun VD. Resting-state functional network disturbances in schizophrenia. Brain Netw Dysfunct Neuropsychiatr Illn (2021), 187–215. doi: 10.1007/978-3-030-59797-9_10

[33] Sen B Mueller B Klimes-Dougan B Cullen K Parhi KK. Classification of major depressive disorder from resting-state fMRI, in: Proc Annu Int Conf IEEE Eng Med Biol Soc EMBS. Berlin, Germany: IEEE. (2019) pp. 3511–4. doi: 10.1109/EMBC.2019.8856453 31946635

[34] Sun Y Collinson SL Suckling J Sim K. Dynamic reorganization of functional connectivity reveals abnormal temporal efficiency in schizophrenia. Schizophr Bull (2019) 45:659–69. doi: 10.1093/schbul/sby077 PMC6483577 29878254

[35] Sendi MSE Zendehrouh E Fu Z Liu J Du Y Mormino E. Disrupted dynamic functional network connectivity among cognitive control networks in the progression of alzheimer’s disease. Brain Connect (2021) 13(6):1–25. doi: 10.1089/brain.2020.0847 PMC10442683 34102870

[36] Sendi MSE Zendehrouh E Miller RL Fu Z Du Y Liu J. Alzheimer’s disease projection from normal to mild dementia reflected in functional network connectivity: A longitudinal study. Front Neural Circuits (2021) 14:593263. doi: 10.3389/fncir.2020.593263 33551754 PMC7859281

[37] Sendi MSE Pearlson GD Mathalon DH Ford JM Preda A Van ETGM. Multiple overlapping dynamic patterns of the visual sensory network in schizophrenia. Schizophr Res (2021) 228:103–11. doi: 10.1016/j.schres.2020.11.055 33434723

[38] Du Y Fu Z Sui J Gao S Xing Y Lin D. NeuroMark: An automated and adaptive ICA based pipeline to identify reproducible fMRI markers of brain disorders. NeuroImage Clin (2020) 28:102375. doi: 10.1016/j.nicl.2020.102375 32961402 PMC7509081

[39] Ellis CA Miller RL Calhoun VD. Neuropsychiatric Disorder Subtyping Via Clustered Deep Learning Classifier Explanations. 45th Annual International Conference of the IEEE Engineering in Medicine and Biology Society (2023) Sydney, Australia: IEEE, 1–4. doi: 10.1101/2022.12.14.520428 38083012

[40] Fu Z Sui J Turner JA Du Y Assaf M Pearlson GD. Dynamic functional network reconfiguration underlying the pathophysiology of schizophrenia and autism spectrum disorder. Hum Brain Mapp (2021) 42:80–94. doi: 10.1002/hbm.25205 32965740 PMC7721229

[41] Sendi MSE Chun JY Calhoun VD. Visualizing functional network connectivity difference between middle adult and older subjects using an explainable machine-learning method, in: Proceedings - IEEE 20th International Conference on Bioinformatics and Bioengineering, BIBE 2020. Cincinnati, USA: IEEE. (2020) pp. 955–60. doi: 10.1109/BIBE50027.2020.00162 33018257

[42] Fu Z Iraji A Turner JA Sui J Miller R Pearlson GD. Dynamic state with covarying brain activity-connectivity: On the pathophysiology of schizophrenia. Neuroimage (2021) 224:117385. doi: 10.1016/j.neuroimage.2020.117385 32950691 PMC7781150

[43] Ellis CA Miller RL Calhoun VD. Identifying neuropsychiatric disorder subtypes and subtype-dependent variation in diagnostic deep learning classifier performance. 45th Annual International Conference of the IEEE Engineering in Medicine and Biology Society. Sydney, Australia: IEEE (2022), 1–4s. doi: 10.1101/2022.10.27.514124 38083012

[44] Macqueen J. Some methods for classification and analysis of multiVariate observations. Proc Berkeley Symp Math Stat Probab (1965) 5:281–97.

[45] Pedregosa F Weiss R Brucher M. Scikit-learn: machine learning in python. J Mach Learn Res (2011) 12:2825–30. doi: 10.48550/arXiv.1201.0490

[46] Allen EA Damaraju E Plis SM Erhardt EB Eichele T Calhoun VD. Tracking whole-brain connectivity dynamics in the resting state. Cereb Cortex (2014) 24:663–76. doi: 10.1093/cercor/bhs352 PMC3920766 23146964

[47] Ellis CA Sendi MSE Geenjaar EPT Plis SM Miller RL Calhoun VD. Algorithm-agnostic explainability for unsupervised clustering (2021). Available online at: http://arxiv.org/abs/2105.08053.

[48] Suganya R Shanthi R. Fuzzy C-means algorithm-A review. Int J Sci Res Publ (2012) 2:2250–3153.

[49] Demircan S Kahramanli H. Application of fuzzy C-means clustering algorithm to spectral features for emotion classification from speech. Neural Comput Appl (2018) 29:59–66. doi: 10.1007/s00521-016-2712-y

[50] Munusamy S Murugesan P. Modified dynamic fuzzy c-means clustering algorithm – Application in dynamic customer segmentation. Appl Intell (2020) 50:1922–42. doi: 10.1007/s10489-019-01626-x

[51] Kahali S Sing JK Saha PK. A new entropy-based approach for fuzzy c-means clustering and its application to brain MR image segmentation. Soft Comput (2019) 23:10407–14. doi: 10.1007/s00500-018-3594-y

[52] Scikit-fuzzy (2022). Available online at: https://scikit-fuzzy.github.io/scikit-fuzzy/.

[53] Fuzzy C-means clustering. In: MATLAB R2022b Available at: https://www.mathworks.com/help/fuzzy/fuzzy-c-means-clustering.html.

[54] van Erp TGM Preda A Turner JA Callahan S Calhoun VD Bustillo JR. Neuropsychological profile in adult schizophrenia measured with the CMINDS. Psychiatry Res (2015) 230:826–34. doi: 10.1016/j.psychres.2015.10.028.Neuropsychological PMC4692593 26586142

[55] Kay SR Fiszbein A Opler LA. The positive and negative syndrome scale (PANSS) for schizophrenia. Schizophr Bull (1987) 13:261–76. doi: 10.1093/schbul/13.2.261 3616518

[56] Cavanna AE Trimble MR. The precuneus: A review of its functional anatomy and behavioural correlates. Brain (2006) 129:564–83. doi: 10.1093/brain/awl004 16399806

[57] Stevens FL Hurley RA Taber KH. Anterior cingulate cortex: Unique role in cognition and emotion. J Neuropsychiatry Clin Neurosci (2011) 23:121–5. doi: 10.1176/jnp.23.2.jnp121 21677237

[58] Leech R Sharp DJ. The role of the posterior cingulate cortex in cognition and disease. Brain (2014) 137:12–32. doi: 10.1093/brain/awt162 23869106 PMC3891440

[59] Preti MG Bolton TA Van De Ville D. The dynamic functional connectome: State-of-the-art and perspectives. Neuroimage (2017) 160:41–54. doi: 10.1016/j.neuroimage.2016.12.061 28034766

[60] Fu Z Du Y Calhoun VD. The dynamic functional network connectivity analysis framework. Engineering (2019) 5:190–3. doi: 10.1016/j.eng.2018.10.001 PMC7265753 32489683

[61] Trauwaert E. On the meaning of Dunn’s partition coefficient for fuzzy clusters. Fuzzy Sets Syst (1988) 25:217–42. doi: 10.1016/0165-0114(88)90189-3

[62] Du Y Fryer SL Fu Z Lin D Sui J Chen J. Dynamic functional connectivity impairments in early schizophrenia and clinical high-risk for psychosis. Neuroimage (2018) 180:632–45. doi: 10.1016/j.neuroimage.2017.10.022.Dynamic PMC5899692 29038030

[63] Breiman LEO. Random forests. Mach Learn (2001) 45:5–32. doi: 10.1023/A:1010933404324

[64] Fisher A Rudin C Dominici F. Model class reliance: variable importance measures for any machine learning model class, from the “Rashomon” Perspective. arXiv Prepr arXiv (2018), 180101489v1.

[65] Ellis CA Miller RL Calhoun VD. A novel explainable fuzzy clustering approach for fMRI dynamic functional network connectivity analysis. 45th Annual International Conference of the IEEE Engineering in Medicine and Biology Society. IEEE (2023) p. 1–4. doi: 10.1101/2023.01.29.526110 38083353

[66] Kendall MG. A new measure of rank correlation. Biometrika (1938) 30:81–93. doi: 10.2307/2332226

[67] Virtanen P Gommers R Oliphant TE Haberland M Reddy T Cournapeau D. SciPy 1.0: fundamental algorithms for scientific computing in Python. Nat Methods (2020) 17:261–72. doi: 10.1038/s41592-019-0686-2 PMC7056644 32015543

[68] Benjamini Y Hochberg Y. Controlling the false discovery rate: A practical and powerful approach to multiple testing. J R Stat Soc Ser B (1995) 57:289–300. doi: 10.1111/j.2517-6161.1995.tb02031.x

[69] Liang S Deng W Li X Wang Q Greenshaw AJ Guo W. Aberrant posterior cingulate connectivity classify first-episode schizophrenia from controls: A machine learning study. Schizophr Res (2020) 220:187–93. doi: 10.1016/j.schres.2020.03.022 32220502

[70] Wada M Nakajima S Tarumi R Masuda F Miyazaki T Tsugawa S. Resting-state isolated effective connectivity of the cingulate cortex as a neurophysiological biomarker in patients with severe treatment-resistant schizophrenia. J Pers Med (2020) 10:1–12. doi: 10.3390/jpm10030089 PMC7564631 32823914

[71] Wang D Zhou Y Zhuo C Qin W Zhu J Liu H. Altered functional connectivity of the cingulate subregions in schizophrenia. Transl Psychiatry (2015) 5. doi: 10.1038/tp.2015.69 PMC4490280 26035059

[72] Guo S Palaniyappan L Yang B Liu Z Xue Z Feng J. Anatomical distance affects functional connectivity in patients with schizophrenia and their siblings. Schizophr Bull (2014) 40:449–59. doi: 10.1093/schbul/sbt163 PMC3932090 24282323

[73] Mashal N Vishne T Laor N. The role of the precuneus in metaphor comprehension: Evidence from an fMRI study in people with schizophrenia and healthy participants. Front Hum Neurosci (2014) 8:818. doi: 10.3389/fnhum.2014.00818 25360101 PMC4199320

[74] Rikandi E Pamilo S Mäntylä T Suvisaari J Kieseppä T Hari R. Precuneus functioning differentiates first-episode psychosis patients during the fantasy movie Alice in Wonderland. Psychol Med (2017) 47:495–506. doi: 10.1017/S0033291716002609 27776563

[75] Aryutova K Paunova R Kandilarova S Stoyanova K Maes MH Stoyanov D. Differential aberrant connectivity of precuneus and anterior insula may underpin the diagnosis of schizophrenia and mood disorders. World J Psychiatry (2021) 11:1274–87. doi: 10.5498/wjp.v11.i12.1274 PMC8717032 35070777

[76] Galindo L Bergé D Murray GK Mané A Bulbena A Pérez V. Default mode network aberrant connectivity associated with neurological soft signs in schizophrenia patients and unaffected relatives. Front Psychiatry (2018) 8:298. doi: 10.3389/fpsyt.2017.00298 29375404 PMC5767074

[77] Li S Hu N Zhang W Tao B Dai J Gong Y. Dysconnectivity of multiple brain networks in schizophrenia: A meta-analysis of resting-state functional connectivity. Front Psychiatry (2019) 10:482. doi: 10.3389/fpsyt.2019.00482 31354545 PMC6639431

[78] Garrity AG Pearlson GD Mckiernan K Lloyd D Kiehl KA Calhoun VD. Aberrant “Default mode” Functional connectivity in schizophrenia. Am J Psychiatry (2007) 164:450–7. doi: 10.1176/ajp.2007.164.3.450 17329470

[79] Forlim CG Klock L Bächle J Stoll L Giemsa P Fuchs M. Reduced resting-state connectivity in the precuneus is correlated with apathy in patients with schizophrenia. Sci Rep (2020) 10:1–8. doi: 10.1038/s41598-020-59393-6 32054907 PMC7018974

[80] Lee H Lee D Park K Kim C Ryu S. Default mode network connectivity is associated with long-term clinical outcome in patients with schizophrenia. NeuroImage Clin (2019) 22:101805. doi: 10.1016/j.nicl.2019.101805 30991621 PMC6451190

[81] Olivetti E Mognon A Greiner S Avesani P. Brain decoding: Biases in error estimation. In: Proc - work brain decod pattern recognit challenges neuroimaging, WBD 2010 - conjunction with theInternational conf pattern recognition, ICPR 2010 (2010) (Istanbul, Turkey: IEEE). p. 40–3. doi: 10.1109/WBD.2010.9

[82] Askari S. Fuzzy C-Means clustering algorithm for data with unequal cluster sizes and contaminated with noise and outliers: Review and development. Expert Syst Appl (2021) 165:113856. doi: 10.1016/j.eswa.2020.113856

[83] Ghosh S Dubey SK. A comparative analysis of fuzzy C-means clustering and K means clustering algorithms. Int J Adv Comput Sci Appl (2013) 4. doi: 10.14569/IJACSA.2013.040406

[84] Sendi MSE Salat DH Miller RL Calhoun VD. Two-step clustering-based pipeline for big dynamic functional network connectivity data. Front Neurosci (2022) 16:895637. doi: 10.3389/fnins.2022.895637 35958983 PMC9358255

