# Supplementary material for: Explainable fuzzy clustering framework reveals divergent default mode network connectivity dynamics in schizophrenia
Source: Front Psychiatry. 2024 Feb 15;15:1165424. doi: 10.3389/fpsyt.2024.1165424 (PMC10941842; doi:10.3389/fpsyt.2024.1165424)
Supplement: Supplementary file 1 [file DataSheet_1.docx]

Supplementary Material

# Clustering Approach Parameters

We initialized the clustering with 100 random seeds and selected the seed from the initialization with the highest fuzzy partitioning coefficient (61). The fuzzy partition coefficient (i.e., Dunn’s partition coefficient) varies from 0 to 1, with 0 indicating that samples have an equal probability of belonging to each class and 1 indicating that clusters are well defined with samples having high probabilities for one cluster. Based on default scikit-fuzzy parameter values, we used a maximum number of iterations of 1000 and decreased the error parameter to 0.0001. Additionally, we optimized the fuzziness parameter, m, selecting from potential values of 1.1, 1.3, 1.5, 2, 2.5, 3.0, 3.5, 4.0, and 4.5 based on their fuzzy partition coefficient values. There are not really established methods for deciding upon an m parameter. However, previous studies have identified appropriate m values as being between 1.5 and 4 (63), so we tried parameter values near that range.

# Description of Explainability Approaches

## G2PC

G2PC is an adaptation of permutation feature importance to the domain of unsupervised clustering explainability. Permutation feature importance was originally developed to explain random forest models (64) and was later extended in a model-agnostic manner to explain a variety of supervised models (65). G2PC involves (1) permuting a particular feature across all samples in a dataset for a given number of repeats, (2) reassigning the perturbed samples to the previously identified clusters, and (3) calculating the percentage of samples in each repeat that switch clusters. Feature importance is considered to linearly increase with the percentage of samples that switch clusters following the perturbation of a feature (i.e., increased sensitivity of the cluster to the perturbation of a feature corresponds to increased importance of that feature). In our analysis, we permute each feature 1,000 times.

## GP2D

A key shortcoming of G2PC is that in a data space with a high number of dimensions perturbing a single feature typically only affects a minority of samples. The use of fuzzy c-means clustering, rather than a hard clustering method like k-means clustering, presents a new opportunity. Specifically, the sensitivity of the clustering to perturbation can be measured as the change in the distribution of probabilities for each sample belonging to each cluster, rather than the percentage of samples that completely switch clusters. As such, our novel approach involves the same first two steps as G2PC. However, we quantify the effect of perturbation by calculating the Kullback-Leibler divergence (KLD) (67) between the original probabilities of a given sample belonging to each cluster and the probabilities for the perturbed sample belonging to each cluster. We repeated the permutation for 1,000 repeats and then calculated two summary metrics from the KLD. (1) We calculated the median KLD across all samples for each repeat (i.e., median GP2D), and (2) we summed the total KLD across all samples for each repeat (i.e., total GP2D). As such, the complete workflow for GP2D involves (1) permuting a particular features across all samples in a dataset for a given number of repeats, (2) obtaining probabilities for the likelihood of the perturbed samples belonging to each of the previously identified fuzzy clusters, (3) calculating the KLD of the effect of perturbation on the cluster probabilities, and (4) calculating summary metrics (i.e., median and total) for the KLD values corresponding to each sample.

## G2PC and GP2D Comparison

We hypothesized that the total GP2D would be more likely to be strongly affected by the select group of samples that would be sensitive to G2PC (i.e, the samples that completely switched clusters), and that the median GP2D metric would differ from both G2PC and total GP2D by accounting for more of the distribution of the effects of perturbation across all samples. To test these hypotheses, we ranked each feature based on its median importance across all 1,000 repeats for each method, producing 3 rankings (i.e., G2PC, median GP2D, and total GP2D). We ranked the median importance value of each feature from greatest to least. We then applied Kendall’s rank correlation (68) in SciPy (69) in a pair-wise approach, comparing G2PC ranks to total GP2D ranks, G2PC ranks to median GP2D ranks, and total GP2D ranks to median GP2D ranks. After obtaining p-values for each test, we applied FDR correction (70) to reduce the likelihood of false positives. We used Kendall’s rank correlation as it tends to be more robust than other rank correlation methods like Spearman’s correlation.

## LP2D

Building upon the GP2D approach, we also calculated the mean of the KLD for each sample across repeats. We used the resulting values for two sets of analyses. (1) We wanted to determine whether our novel P2D approach was actually able to capture the effects of perturbation upon more samples than G2PC, so we calculated the percentage of samples that had non-zero KLD values. (2) We wanted to use LP2D as an approach for estimating the stability of individuals to each dFNC feature. To that end, we also used the standard deviation of the mean of LP2D values across samples for each participant as dynamical features (i.e., for each sample take the mean KLD across repeats and then take the standard deviation of the resulting values across samples belonging to each subject). These stability features and the specific analyses performed upon them will be further discussed in subsequent sections.

# Description of Dynamical Feature Extraction

## Traditional Features

We extracted two types of traditional features: the occupancy rate (OCR) and number of state transitions (NST). These features have been used in many previous studies (1,5,36,37). Extracting these features required thresholding the probabilities of each sample for each fuzzy cluster such that each sample was assigned to the cluster for which it had the highest probability of belonging. The OCR is the percentage of time points that each participant spends in each state, so with 5 dFNC states there are 5 OCRs for each participant. The NST is the number of times that each participant changes states.

## KLD and Entropy-based Features

The first set of novel dynamical features that we introduced used Kullback-Leibler divergence and Shannon entropy. For the KLD-based features, we calculated the KLD of the 5 fuzzy state probabilities between each of the 124 consecutive time steps for each participant. This yielded an array of KLD values between each time point with which we calculated a number of additional values. For each participant, we calculated the mean KLD across time points, the median KLD across time points, the maximum KLD between any two consecutive time points, the range of KLD values between time points, and the KLD between probabilities for the first and last time points in a recording. The KLD-based features quantified different aspects of how the distribution of probabilities across fuzzy states shifted over time. However, we also wanted to gain insight into how the probabilities of each individual fuzzy state shifted over time. To this end, we calculated the Shannon entropy across all time points for each fuzzy states separately (i.e., yielding one entropy value per state).

## Descriptive Statistic-based Features

We next used a number of traditional descriptive statistics as novel dynamical features. Specifically, for each state, we calculated the mean, variance, and range of probabilities across time points for each participant. This yielded 15 features (i.e., 1 mean, 1 variance, and 1 range feature per state). The mean value gave an approximation of how strongly a particular participant resides in a particular fuzzy state. Similar to the Shannon entropy feature, the variance value gives insight into how much the similarity of the dFNC features for each participant varies over time. Lastly, whereas the mean feature would capture how strongly a participant resided within a given state and the variance feature would capture how the similarity of a participant to each fuzzy state varied over time, the range feature sought to give insight into the more extreme probabilities for each time-series that might otherwise be obscured. The utility of this feature makes sense given that previous studies have identified the effects of SZ upon the brain to be highly localized (24).

## Correlation-based Features

We also wanted to identify relationships between specific states. As such, we calculated the correlation between the probabilities for each state over time. This yielded 10 features. The use of correlation could give insight into whether probabilities shifting from a particular state over time are redistributed to a specific alternative state or redistributed to a number of states.

## Cumulative Difference-based Features

Similar to how previous studies have sought to understand the total distance traveled within a particular state (11), we wanted to understand in an absolute manner how much the probabilities of a participant being in a state change over time. As such, we calculated the difference between probabilities for each state across each pair of consecutive time points and summed the absolute differences for each state. This yielded 5 features (i.e., 1 feature per state).

## Uniformity-based Feature

Lastly, we sought to quantify the degree to which state probabilities were uniformly distributed across states. To this end, we calculated the absolute difference between the 5 state probabilities at each step and the uniform probability of 0.2. We then summed the absolute differences at each step and averaged across time steps for each participant.

# LR-ENR Parameter Selection

When applying 10-fold nested cross-validation, 64%, 16%, and 20% of the data being assigned to training, validation, and test sets, respectively. Within the inner cross-validation folds, we optimized the ratio of L1 to L2 normalization, selecting from values of 0.25, 0.5, 0.75, 0.85, 0.9, 0.95, and 0.99. We further optimized the inverse of the regularization strength, selecting from 100 values geometrically spaced between 10^-4^ and 10^4^. We used the saga solver from Scikit-Learn (45), and allowed a maximum of 200,000 iterations. In the inner folds, we maximized the model accuracy. After obtaining the optimal parameter set for each inner fold, we retrained each model on both training and validation data using the optimal parameters before testing.

# Supplementary Figures

#
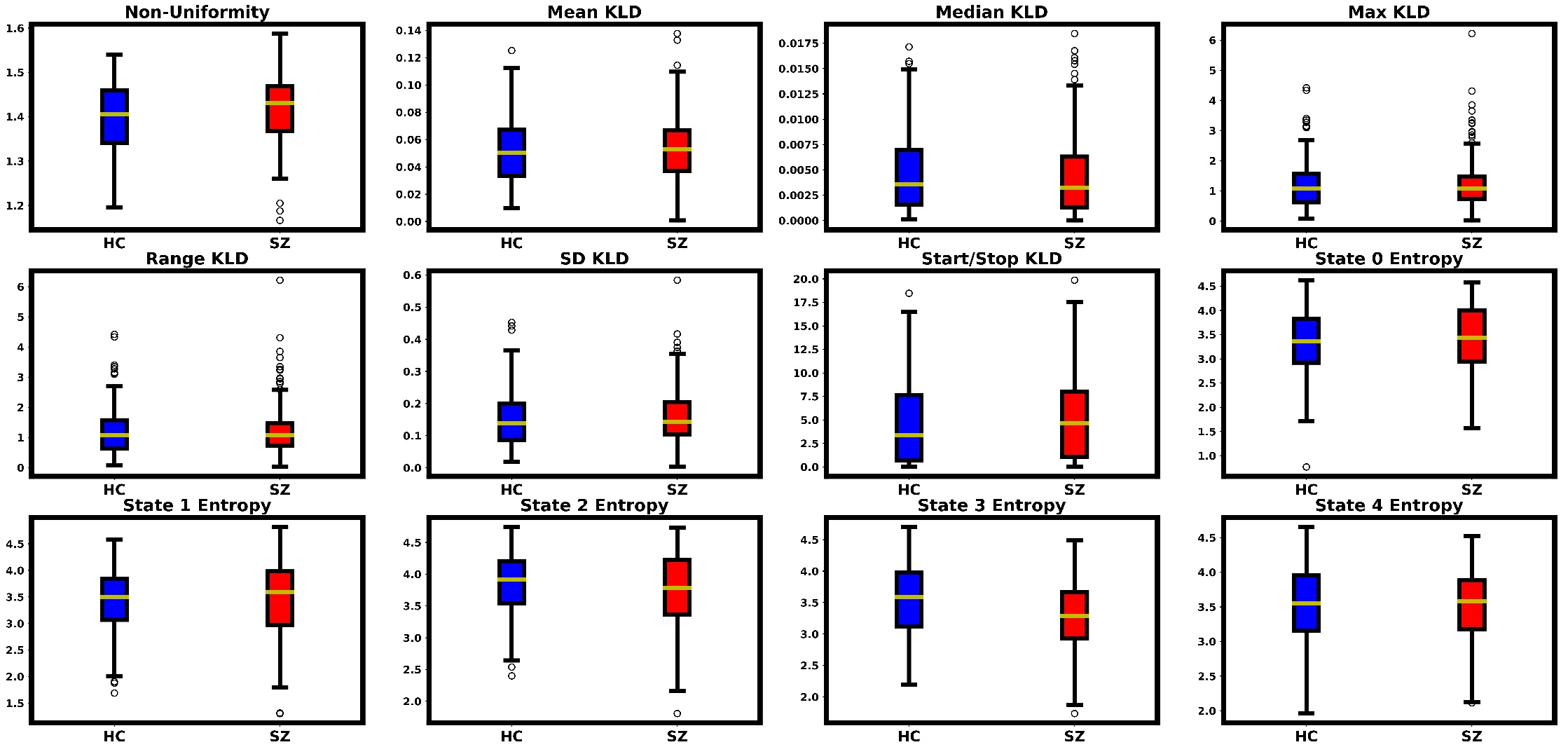
Supplementary Figure 1. Boxplots of Non-Uniformity, KLD, and Entropy Features. Each panel shows boxplots for the dynamical features for HCs (left) and SZs (right). The title of each panel indicates the dynamical feature included in the boxplots.


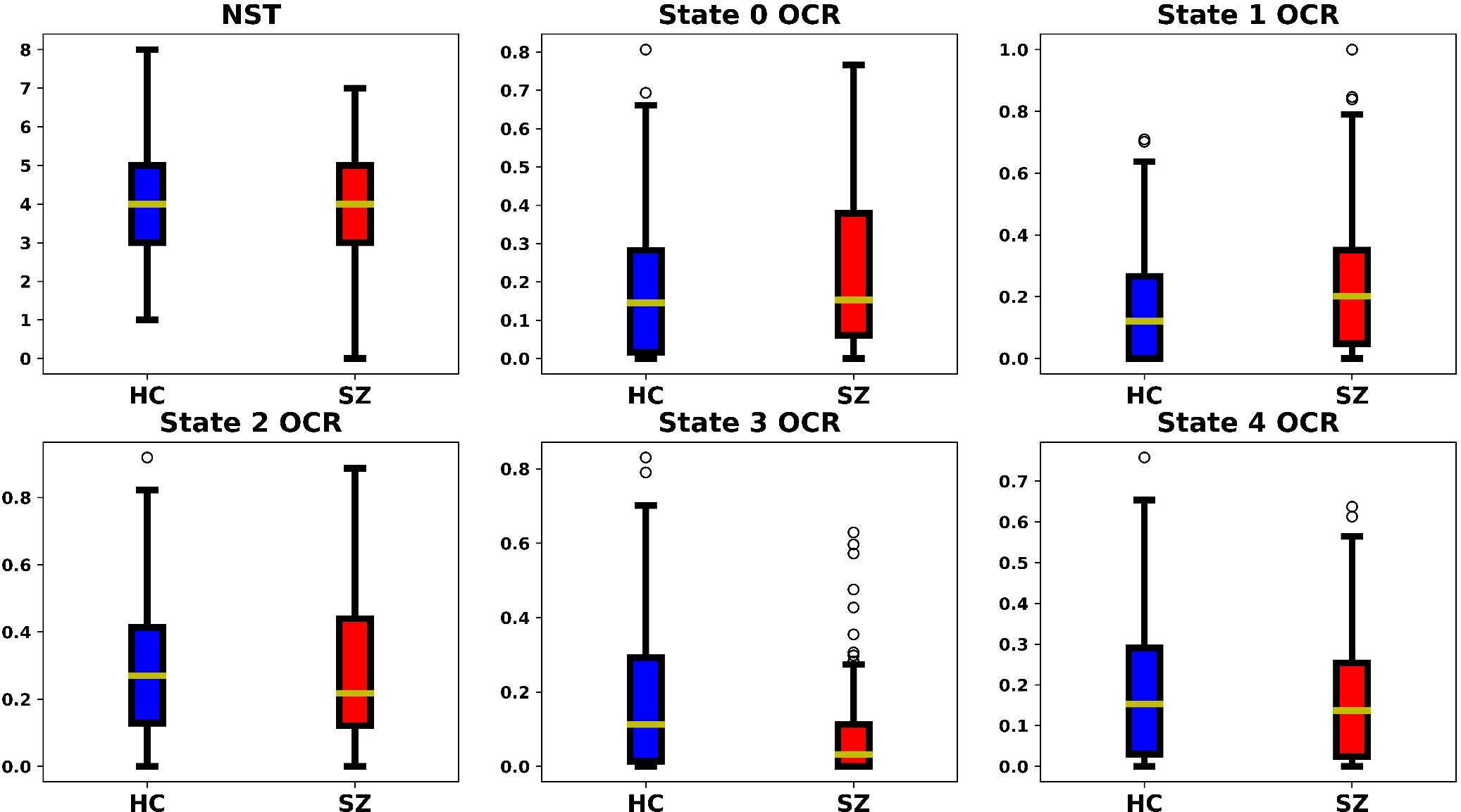


**Supplementary Figure 2.** Traditional NST + OCR Features. Each panel shows boxplots for the dynamical features for HCs (left) and SZs (right). The title of each panel indicates the dynamical feature included in the boxplots.


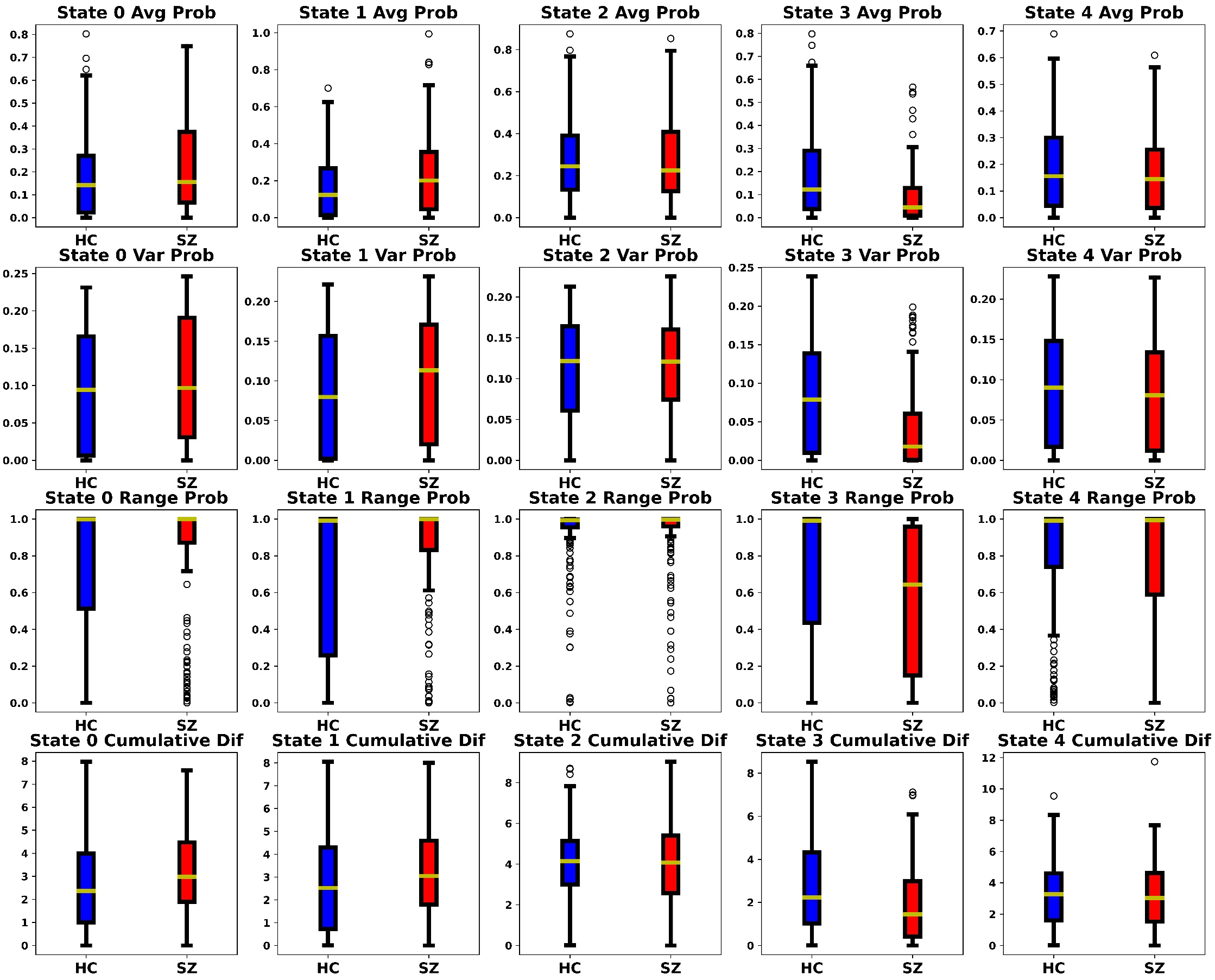


**Supplementary Figure 3.** Boxplots of Average, Variance, Range, and Cumulative Difference Features. Each panel shows boxplots for the dynamical features for HCs (left) and SZs (right). The title of each panel indicates the dynamical feature included in the boxplots.


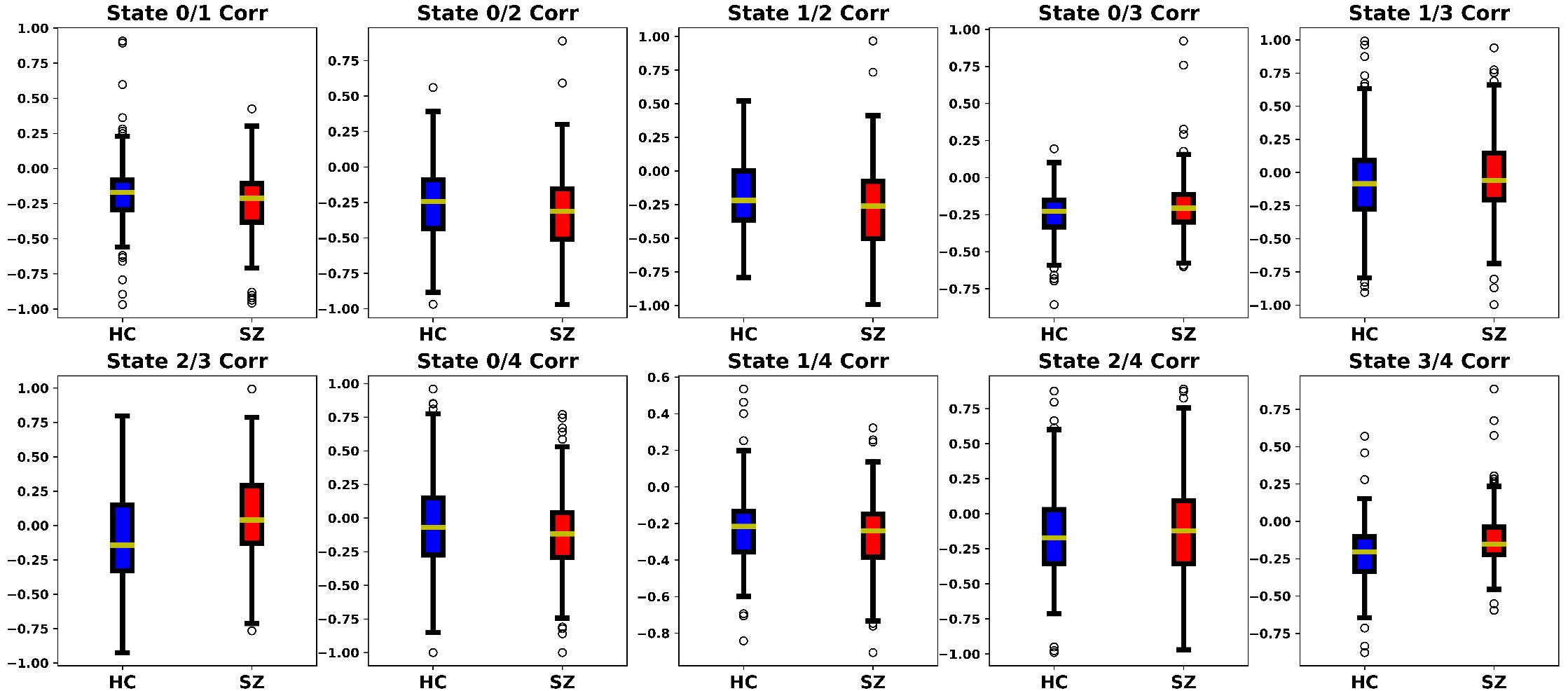


**Supplementary Figure 4.** Boxplots of Correlation Features. Each panel shows boxplots for the dynamical features for HCs (left) and SZs (right). The title of each panel indicates the dynamical feature included in the boxplots.
